# The Current Status of Cytomegalovirus (CMV) Prevalence in the MENA Region: A Systematic Review

**DOI:** 10.3390/pathogens8040213

**Published:** 2019-10-31

**Authors:** Hassan Al Mana, Hadi M. Yassine, Nadin N. Younes, Anjud Al-Mohannadi, Duaa W. Al-Sadeq, Dalal Alhababi, Elham A. Nasser, Gheyath K. Nasrallah

**Affiliations:** 1 Department of Biomedical Science, College of Health Sciences, QU Health, Qatar University, P.O. Box 2713 Doha, Qatar; h.almana@qu.edu.qa (H.A.-M.); hyassine@qu.edu.qa (H.M.Y.); ny1204022@student.qu.edu.qa (N.N.Y.); aa1003879@student.qu.edu.qa (A.A.-M.); da1206066@qu.edu.qa (D.W.A.-S.); 200153669@student.qu.edu.qa (D.A.); 200651385@student.qu.edu.qa (E.A.N.); 2 Biomedical Research Center, Qatar University, P.O. Box 2713 Doha, Qatar

**Keywords:** cytomegalovirus, transplantation, transfusion, congenital, MENA region

## Abstract

Human cytomegalovirus (CMV) is a highly prevalent herpesvirus worldwide. According to the Centers for Disease Control and Prevention (CDC) and the World Health Organization (WHO), CMV infects people of all ages, and by the age of five, approximately one-third of children in the United States are infected. Although the infection is generally asymptomatic, it can cause severe disease in immunocompromised patients, transplant and transfusion recipients, as well as newborn neonates. The objective of this study is to systematically review published literature on CMV in the MENA region to estimate its incidence in the region and describe its epidemiological and clinical significance. The literature was searched through four scientific databases: PubMed, Scopus, Science Direct, and Web of Science. A total of 72 studies from 11 countries satisfied the inclusion criteria, covering a period from 1988–2019. The CMV IgG seroprevalence ranged from 8.7–99.2% (SD = 38.95%). CMV incidence in these countries ranged between 1.22% and 77% in transplant and transfusion recipients, with an increase in incidence with advanced age. However, the incidence rate was unclear for congenital CMV due to the variability of the reporting. This review highlights the need for more robust and well-designed studies to better estimate CMV incidence in the MENA region, standardize diagnostic criteria, and consider prophylactic and pre-emptive treatments to limit the morbidity and mortality of the disease.

## 1. Introduction

Cytomegalovirus (CMV) is a global herpesvirus that is highly prevalent worldwide. It is a ubiquitous virus with a prevalence of about 100% in both Africa and Asia, and 80% in Europe and North America [1]. It is a member of the family *Herpesviridae* and genus *Cytomegalovirus* [2]. CMV is classified as a β-herpesvirus (HHV-5) and considered to be the largest herpesvirus to infect humans, with a genome of nearly 240 kb [3]. According to the Centers for Disease Control and Prevention (CDC) and World Health Organization (WHO), CMV can infect people of all ages; over 50% of adults are infected with CMV by the age of 40, and approximately one in three children are infected with CMV by the age of five in the United States [4]. Although CMV infection is usually asymptomatic, it could lead to severe outcomes in immunosuppressed individuals, particularly transplant recipients and blood transfusion patients [5]. CMV disease can mimic a range of different manifestations and pose significant diagnostic challenges, leading to late or inaccurate diagnosis and adverse health outcomes [6]. Therefore, CMV infection is considered a critical health concern for high-risk populations.

Since CMV is a persistent latent virus, it can manipulate and evade the immune system. Studies have reported multiple genes in CMV that are responsible for immune evasion that profoundly interfere with both the innate and adaptive immunity of the host, thus preventing viral elimination [7]. Nevertheless, following primary infection, CMV and the host’s immune system reach a homeostatic balance, where a lifelong latency is established primarily in cells of the myeloid lineage [8]. Although reactivation phases can occur, their detection is rare in immunocompetent individuals. The continuous commitment of the immune system to control the viral infection throughout the host’s life places a heavy burden on the host’s immune system, which could lead to vascular diseases and immune senescence in the elderly [9]. Significant suppression of the host immune responses against CMV can alter the life-or-death immune surveillance balance, allowing CMV reactivation or primary infection to cause clinical manifestations [10,11,12,13,14]. Such cases could be observed with transplantation patients that need to undergo immune suppression therapy. CMV is one of the most frequently reported opportunistic viral pathogens in immune-deficient patients, including solid organ transplant (SOT) and hematopoietic stem cell transplant (HSCT) recipients [15]. The uncontrolled viral replication and dissemination can be life-threatening, as it could result in end-organ damage [1]. Furthermore, CMV is also a leading cause of congenital diseases worldwide, resulting in about 29 congenital conditions as reported from the United States alone [16].

Notably, there are significant differences in terms of CMV seroepidemiology between different populations and ethnic backgrounds. Since CMV epidemiological studies are scarce in the MENA region, it was of interest to conduct a systemic review to paint a picture of the current status of CMV and evaluate its incidence rate in high-risk sub-populations in the MENA region, namely transplant and transfusion recipients and the newborns.

## 2. Methods

### 2.1. Search Strategy

We conducted a systematic review of all literature published on CMV in the MENA region using four databases: PubMed, Scopus, Science Direct, and Web of Science. The search covered all literature within the databases up to March 2019. The databases were queried with the keywords: “CMV”, “Cytomegalovirus”, “human herpesvirus 5”, and “HHV-5”. Each keyword was queried individually with the name of each of the countries in the MENA region. The countries considered as part of the MENA region in this review were: Algeria, Bahrain, Djibouti, Egypt, Iran, Iraq, Israel, Jordan, Kuwait, Lebanon, Libya, Morocco, Oman, Palestine, Qatar, Saudi Arabia, Sudan, Syria, Tunisia, United Arab Emirates, and Yemen. In addition to the names of the countries, the terms “west bank”, “Gaza”, “UAE”, “United Arab Emirates”, “Emirates”, “KSA”, “Middle East”, “North Africa”, and “MENA” were used to account for alternate names and ensure complete coverage of the region. PubMed, Science Direct, and Web of Science were searched without filters. Scopus was searched while filtering out books, book chapters, and commentaries. All retrieved citations were imported into EndNote X8, and duplicates were removed using the EndNote X8 built-in “Find Duplicates” feature. Finally, the titles and abstracts of the remaining citations were screened to remove any irrelevant articles, such as plant or animal viruses and human viruses other than CMV.

### 2.2. Study Selection

**Figure 1:**
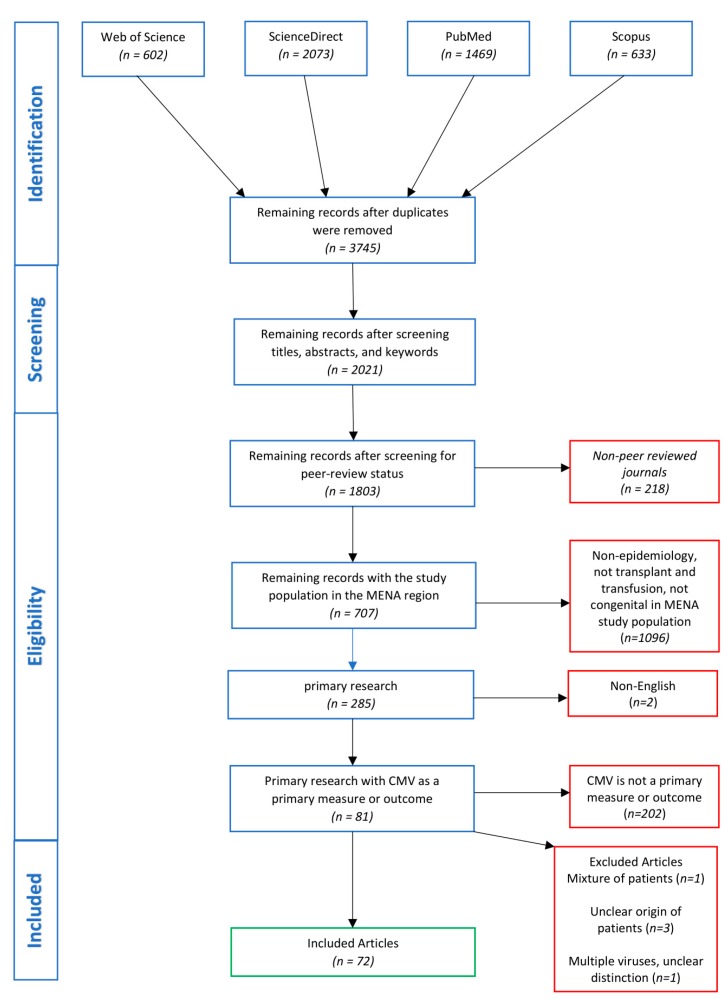
Flow diagram of the search strategy and article selection.

The following inclusion criteria were used in study selection: (i) published in a peer-reviewed journal, (ii) articles studying the epidemiology CMV in the MENA region, (iii) published as primary research articles, (iv) articles published in English or with an abstract in English, and (v) articles in which CMV is a primary measure or outcome of the study. The peer-review status of the published studies was confirmed using Ulrich’s Periodicals Directory (https://www.ulrichsweb.com/). Only studies that satisfied all five inclusion criteria were included in this review. Studies were excluded from the review if: (i) the study consisted of a mixture of patients from within and without the MENA region, and it was not possible to differentiate the data, (ii) it is not clear to differentiate the origins of the patients, and (iii) the study investigated multiple viruses, including CMV, and the data could not be differentiated. A schematic of the search strategy and study selection process is shown in Figure 1.

### 2.3. Data Extraction and Analysis

The studies included in this systematic review were analyzed three times by the same individual to ensure accurate capture of the information. The analyzed data included the country of the study, period of study, number of subjects, type of the study, incidence/prevalence of CMV antibodies or infection, symptoms and complications, proposed risk factors and routes of transmission, as well as treatment and prevention.

## 3. Results

### 3.1. Search Findings

The search yielded 4777 studies, of which 3745 citations remained after removing duplicates (Figure 1). After screening the titles, abstracts, and keywords, 1724 citations were excluded. The removed citations included irrelevant studies, such as those on animal and plant viruses. The remaining 2021 citations were screened against the eligibility criteria. Of these, 218 studies were removed due to the lack of peer-reviewing, and 1096 were removed for not discussing the epidemiology of CMV in transplant and transfusion patients nor its congenital illness. Furthermore, 422 non-primary research studies were removed (i.e., review articles, editorials, communications, case-reports, etc.), two for being published in languages other than English with no English abstract, and 202 for not having CMV as a measure or outcome of the study. One study was excluded as it was retracted by the authors. Finally, five studies were excluded as they met the exclusion criteria. The remaining 72 studies covered 11 countries (Egypt, Iran, Iraq, Israel, Jordan, Kuwait, Oman, Palestine, Saudi Arabia, Sudan, and Tunisia) over 31 years, from 1988–2019. The highest number of studies was from Iran (*n* = 27), followed by Israel (*n* = 21). No studies meeting the eligibility criteria were found from Algeria, Bahrain, Djibouti, Libya, Morocco, Qatar, Syria, the United Arab Emirates, and Yemen.

### 3.2. Epidemiological Findings

The reviewed studies covering CMV incidence in transplant recipients included renal transplants (*n* = 15), hematopoietic stem cell transplant (HSCT; *n* = 4), bone marrow transplants (BMT; *n* = 3), liver transplants (*n* = 3), and coronary artery bypass graft (*n* = 1). Country-wise, the majority of the studies were from Iran (*n* = 19). The remaining studies were published from Saudi Arabia (*n* = 4), Egypt (*n* = 2), Israel (*n* = 2), Kuwait (*n* = 2), Iraq (*n* = 1), Jordan (*n* = 1), Oman (*n* = 1), Sudan (*n* = 1), and Tunisia (*n* = 1). Overall, CMV infection among transplant recipients has been reported with an incidence ranging from 1.22% to 72%, regardless of prophylactic treatment. On the other hand, the anti-CMV IgG seroprevalence rate ranged between 8.7% and 99.2% (SD = 38.95), being mostly reported in Iran (Table 1). Fewer studies reported the IgM seroprevalence (*n* = 3), which ranged from 1.6% to 9.6%. The design and criteria to describe CMV disease in these studies were inconsistent (Table 1). Furthermore, multiple studies reported an association between increased incidences of CMV and advanced age. Other risk factors that were found include anti-rejection therapy, previous exposure to CMV, serological mismatch between the donor and recipient, as well as, several immunological determinants. However, there was a lack of CMV epidemiological studies from several countries in the region (Algeria, Bahrain, Djibouti, Lebanon, Libya, Morocco, Palestine, Qatar, Syria, the UAE, and Yemen), rendering the actual incidence of CMV across the region undetermined. As for congenital CMV, the studies were also inconsistent with their diagnosis and reporting of the results. Therefore, accurate incidence/prevalence data could not be deduced. Furthermore, the majority of the studies report infected cases with no incidence/prevalence data. The 38 studies with epidemiological information included in this review came from Israel (*n* = 19), Iran (*n* = 9), Iraq (*n* = 2), Egypt (*n* = 2), Kuwait (*n* = 1), and Oman (*n* = 1).

## 4. Discussion

To the best of our knowledge, this is the first systematic review study that investigated the epidemiology and status of CMV in immunocompromised patients in the MENA region. A total of 72 out of 3745 screened studies, covering 11 countries, were examined transplantation and blood transfusion recipients as well as the congenital CMV.

### 4.1. CMV in Transplantation Recipients in the MENA Region

CMV infects up to 60–100% of people in adulthood, and it is one of the main agents involved in infectious complications after transplantation [17]. It threatens the survival of transplant recipients and the function of the transplanted organ. Graft rejection and graft-versus-host disease (GVHD) are multisystem disorders that are common complications of transplantation. GVHD occurs when immune cells transplanted from a non-identical donor (the graft) recognize the transplant recipient (the host) as foreign, thereby initiating an immune reaction that causes disease in the transplant recipient [18]. A less well-established risk factor for GVHD is the CMV status of the donor and host [19,20,21,22,23]. Fortunately, it rarely results in mortality, with only one study reporting severe effects [20]. Generally, infection with CMV results in systemic viral syndrome, as shown in Table 1. The main manifestations are characterized by fever, malaise, vomiting, leukopenia, thrombocytopenia, and elevated liver enzymes. Upper digestive tract symptoms and pain are also common [23,24,25,26]. Furthermore, respiratory illnesses, including pneumonia, have been reported in a few studies [24,27,28,29]. Few studies reported other illnesses including hepatitis [25], urinary tract infection [24], rhinitis [28,29], skin conditions [23,24], and rarely, aortic plaques [30]. The disease caused by post-transplant CMV occurs due to the transplantation of an infected organ, reactivation of latent infection, or after primary infection in seronegative transplant patients [31,32]. The occurrence of disease caused by CMV in transplanted patients varies according to the organ transplanted, the serological match between recipient and donor, the immunosuppressive drugs used, and, most importantly, the interference of any additional diseases [17]. The incidence of CMV infection is 50% to 75% in patients undergoing heart-lung or lung transplantation and 50% in patients undergoing pancreas or kidney-pancreas transplantation. The incidence of CMV infection is 9% to 23% after heart transplantation, and 22% to 29% after liver transplantation [33]. In addition, 30% of patients undergoing allogeneic hematopoietic stem cell transplantation (HSCT) and approximately 5% of patients undergoing autologous HSCT develop CMV disease [34]. Moreover, a study on CMV infections in the context of HSCT showed that 68% of pediatric patients who received umbilical cord blood transplantation were CMV seropositive [34].

Multiple studies investigated the association between the risk of CMV infection in transplant patients’ and their demographics, including age, gender, and source of transplantation; however, results were inconsistent. A recently published study from Iran reported that CMV was diagnosed in 178 out of 725 (24.6%) kidney recipients, and showed that the incidence of CMV disease in kidney transplant patients within the age group 41–60 was four-fold more compared to those under 20 years old [27]. Similarly, a study conducted on HSCT in Iran revealed a positive correlation between the age of the recipients and CMV antigenemia [23]. On the other hand, other studies, such as in Saudi Arabia, failed to show this correlation [35]. These observations, along with those from other countries around the globe [36], indicate that age was a risk factor for CMV infection, especially in transplant patients [37,38]. For instance, a study reported that the risk of death was significantly increased in patients >38 years old, who underwent transplantation with peripheral blood, with an unrelated or mismatched donor, and who developed a CMV infection [39]. In addition, a study conducted on 200 allografted patients showed that recipients aged over 16 years were found to be at significant risk of CMV reactivation compared with younger patients (*p* = 0.007) [40].

In addition to age, several other factors have been identified in association with CMV incidence in SOT and HSCT recipients. One of the most significant risk factors is the extensive use of immunosuppressive drugs for patients undergoing organ transplantation. For instance, Taherimahmoudi et al. suggested that immunosuppression therapy using lymphocyte-depleting antibodies, namely anti-thymocyte globulin (ATG) therapy, is considered as one of the leading risk factors for CMV disease [41]. Charfeddine et al. reported that 12 out of the 16 patients who received kidney transplants suffered from CMV infections. Additionally, 6 out of those 12 patients developed acute rejection episodes due to CMV infection after administrating additional immunosuppressive treatment [21]. Such high frequency of infection was attributed to intensive antirejection therapy (including azathioprine, prednisone, cyclosporine, and ATG), among other factors such as previous exposure to CMV prior to transplantation [42], serological mismatch between both donor and recipient (e.g., CMV-negative recipients (R−)) received grafts from CMV-positive donors (D+)), and immune system factors.

Several immune correlates were identified as predictors for post-transplant CMV infection. One of them is mannose-binding-lectin (MBL), an innate humoral immunity protein that is important for pathogen opsonization and activation of complement pathways. One study documented a positive correlation between the infection and low levels of MBL. MBL deficiency compromises the innate immune response, resulting in a higher risk of developing post-transplant CMV disease and increasing the necessity of prophylactic treatment [43]. Other factors include CD 56^+^ T-cell levels, HLA mismatch, and GVHD. CD56^+^ T-cells (also known as NK-like T cells or cytokine-induced killer cells) have a potent cytotoxic effect on CMV, and a significant increase in their levels in renal transplant patients is suggestive of current CMV infection [44]. As for HLA, CMV disease was found to be influenced by more than one HLA allele mismatch [45]. Moreover, some studies have shown that specific alleles can promote or protect from post-transplant CMV disease. For instance, Khalifa et al. reported that 33.3% of CMV pp65 antigenemia positive patients in SOT have the HLA-A * 02 genotype, while patients with HLA-A * 01 (57.1%) had a protective effect against CMV infection [46]. Another study showed that kidney recipients with the HLA-B44 allele are more susceptible to CMV infection after transplantation, while carriers of the HLA-B8 allele are naturally protected from CMV infection [47]. Furthermore, a study showed that specific single-nucleotide polymorphisms (SNPs) in the loci of co-stimulatory molecules CTLA4 and CD28, which function in the regulation of T-cell activation, influence active CMV infection in kidney transplant patients [48]. Overall, these studies suggest the use of HLA typing as a predictor for CMV infection within the context of personalized medicine for prophylactic post-transplantation treatment.

The high risk of CMV infection and transmission through organ transplantation is universal. The prevalence across the globe ranges from 45% in developed countries to near 100% in developing countries [1]. The health burden of CMV infection and its manifestations seen in the MENA region is similar to that seen in other populations [49,50]. Even though the risk is high for transplant patients in the MENA region, only a few studies suggested screening for CMV in donors. For instance, in Saudi Arabia, screening for CMV and other pathogens before transplantation, especially in HSCT, was found to improve patient safety and mitigate the risk of accidental CMV infection [51]. One of the suggested approaches to protect from post-transplant CMV infection is the treatment with antibodies targeting CMV antigens (pp. 28, 150), which was shown to be effective in kidney transplantation [52]. On the other hand, Shibolet et al. demonstrated the development of late CMV disease and the occurrence of rejection episodes in liver transplantation recipients, regardless of the early use of antiviral prophylaxis [29]. In conclusion, due to the potential abnormalities associated with CMV infection and various morbidities, the establishment of preventive measures, especially vulnerable populations, including transplant recipients, is required.

### 4.2. CMV in Blood Transfusion Recipients in the MENA Region

CMV infection not only compromises transplantation but can also compromise the effectiveness of blood-transfusions, leading to transfusion-transmitted CMV infection, especially in immunocompromised patients. Transfusion-transmitted CMV infection occurs mainly due to the re-activation of the latent virus in WBCs. Many studies were performed to assess the seropositivity rates in different MENA region countries. Only a few studies, conducted mainly in Iran and Egypt, reported CMV infection after blood transfusion in the MENA region. The type of transfusion mainly discussed by the articles included in this review is whole blood transfusion; this is because most of the patients that need regular and frequent whole blood donations are anemic and thalassemic patients such as transfusion of other blood products like plasma transfusion, which is safer since most of the time it is autologous. That is why all research done on CMV seroprevalence was about patients with thalassemia who received multiple types of blood from different donors that could have been positive for the virus. A recent study in Mashhad, Iran, showed that out of 1008 blood samples that were tested for CMV antibodies, 99.2% were found to be positive [53]. In another study from Iran, Sepehrvand et al. reported that the high incidence of CMV antibodies that were present in transfusion patients’ blood is a result of receiving blood from CMV infected donors [54].

Furthermore, Mahmoud et al. showed that frequent blood transfusions among thalassemic children in Upper Egypt exposed them to a higher risk of transfusion-transmitted infections, including CMV. In this study, high rates of CMV infections were reported in children receiving a blood transfusion, and the infection was positively correlated with increasing age and the duration of the thalassemia [55]. These high rates of CMV infections are attributed to the high CMV seroprevalence among blood donors in Egypt, as reported by Gawad et al. In their study, 96.6% of blood donors (out of 88 tested blood samples) were CMV seropositive [56].

Overall, these findings demonstrate a high rate of prevalence of CMV infections in the MENA region, and as such, posing serious implications for the blood transfusion practices if proper screening measures are not implemented. High CMV positivity in transfusion patients, highlighted clearly in some of MENA countries (Table 1), assures that there is a crucial need for mitigation plans to reduce CMV transmission in transfusion patients.

### 4.3. Congenital CMV (cCMV) in the MENA Region

The reviewed studies (*n* = 38) documented a total of 1271 fetuses and infants from eight countries. The subjects were either confirmed CMV cases with vertical transmission of the virus or unconfirmed seropositive cases [71,72,73,74,75,76,77]. Clinical CMV manifestations were assessed either with antenatal ultrasound or postnatal cCMV associated complications. The majority of the studies came from Israel (*n* = 19), where the primary clinical features included abnormal ultrasound, severe brain dysmorphology (mainly in the first and second trimesters), hearing abnormalities, as well as occult CNS symptoms that are associated with neurologic sequelae, abortion, premature deaths, and congenital malformations [71,74,78,79,80,81,82,83,84,85,86]. On the other hand, some studies reported asymptomatic cCMV infections [87,88].

In terms of disease morbidity, one of the common complications of cCMV observed in the MENA region is abnormal brain sonography. Hadar et al. reported a 67% occurrence of abnormal brain sonography in infants born to mothers with primary maternal infection, and 8.3% in infants born to mothers with non-primary infection [89]. Another common complication of cCMV is hearing loss. A retrospective study in Israel correlated early cCMV infection with hearing loss [79]. In addition, cCMV could lead to hepatic damage, including hepatitis and cholestatic disease [88]. The frequency of which (6.6%) was found to be less than was previously expected and is far out shadowed by CNS involvement (84.6%) and hearing loss (53.8%) [88]. In all cases, however, rapid antiviral treatment showed improvement in symptoms, albeit over prolonged periods [79,88]. As for mortality, higher rates were found in association with primary cCMV infections compared to recurrent infections, and abortions were reported at all pregnancy stages [73,90]. Jahromi et al. found that there is an association between seropositivity and abortion rate [91].

Nevertheless, there is substantial variability in the definitions of symptomatic and asymptomatic cCMV infection, study designs, and the methods of determining CMV infection. This variability, along with the variability in study design and detection methods, created difficulty in assessing the overall status of cCMV in the region (Table 2). Similar variations are also seen in the US and Germany, suggesting complicated disease manifestations, depending on the genetic, health, and nourishment status of the infants and their mothers [92,93,94,95]. Still, the incidence rate of cCMV in the MENA region seems to be higher than in other populations. Schlesinger et al. reported an incidence of 945/135,000 (0.7%) births in 2005 in Israel [96,97]. This incidence rate is similar to that found in the United States (0.6–0.7%) [98]. The rate is, however, higher than in Europe, to which Israel’s healthcare system is more similar. For instance, Sweden and the UK have incidence rates of 4.6/1000 births (0.46%) and 3.2/1000 births (0.32%), respectively, and a decades-long prospective study in Denmark found the incidence rate to be 0.4% [99,100].

### 4.4. Laboratory Diagnosis of CMV in the MENA Region

CMV diagnosis was traditionally performed by serologic testing and viral cultures from multiple samples; however, molecular diagnosis is currently the standard [116]. A similar trend can be seen in the MENA region with the diagnosis of CMV. In the 1980s, culture and serological methods were used to detect CMV. The trend moved towards molecular testing, with polymerase chain reaction (PCR) becoming more prominent over time. In the reviewed literature, multiple diagnostic laboratory tools were used, including serology, PCR, antigenemia assays, immunohistochemistry, and culture.

Serological methods indirectly provide evidence of current or prior infection by detection of antibodies in serum. The presence of anti-CMV IgM antibodies can be used to diagnose a recent or acute infection, or at least a fourfold increase in IgG antibody titer in subsequent specimens obtained two to four weeks apart [116]. However, IgM antibodies can persist for several months, leading to false-positive results for acute infection [117]. Thus, a definite diagnosis of CMV infection cannot be obtained from serological tests. Nevertheless, serological antibody detection remains a cost-effective screening tool. Serological methods alone, or in conjunction with other methods, are employed in the MENA region to screen for CMV before and after transplantation [23,47,52,57,63,68,70,118,119,120,121,122,123,124]. Moreover, serology is used in Israel to screen pregnant women for CMV infection. Although no formal regulations exist, still, most hospitals perform routine screening for anti-CMV antibodies using the criteria mentioned earlier to establish a diagnosis [74,125,126]. Once the presence of CMV is established in the mother, congenital CMV is diagnosed either by pre-natal amniocentesis followed by culture or PCR, or by PCR or antigenemia assays in the urine of the infant [73,75,76,78,80,104,108,127].

In recent years, PCR has become prominent in CMV testing in the MENA region. PCR is highly sensitive and specific. However, its main drawback is that it cannot differentiate between latent and active virus [116]. Additionally, there is variability in the viral loads obtained by different laboratories for the same specimen. In response, the WHO developed an international quantitative standard to standardize measurements and facilitate studies to correlate viral load with the development of the disease [128,129]. Nevertheless, the variability among assays continues to exist due to differences in specimen types, DNA extraction methods, as well as the PCR protocol [130,131,132]. Currently, PCR is commonly used in the MENA region for the diagnosis of CMV. It is used to test for congenital CMV in amniotic fluid or urine [73,75,76,78,80,104,108,127], to monitor CMV infection after transplant [41,62,133,134,135,136], as well as end-organ testing when a specific organ is affected, such as HIV associated CMV retinitis [137].

Finally, CMV antigenemia, immunohistochemistry, and culture methods are used in the MENA region as a definite diagnosis of CMV infection. Antigenemia is used in the diagnosis of congenital CMV and the monitoring of viremia in transplant patients. The assay is based on the detection of the CMV pp65 protein in peripheral blood leukocytes. Their advantage over measuring anti-CMV antibodies and PCR is that it can detect the active virus, correlating the result of the assay with viremia [116]. Immunohistochemistry assays use labeled antibodies directed towards CMV antigens (early antigen, immediate early antigens, pp65, and late antigen) and are visualized under the microscope. A positive result in immunohistochemistry indicates the presence of CMV [138]. In the MENA region, this method was only used in studies of tissue biopsies of invasive CMV infections in the case of cancer [139,140,141,142]. This usage is in line with the international consensus for the diagnosis of CMV in invasive tissue disease [138]. As for culture, conventional methods have been replaced by the more rapid shell-vial cultures [116]. Shell-vial culture involves the centrifugation of a patient specimen onto a cell monolayer in a vial, which speeds up the time to the result. The virus is then detected in the monolayer using fluorescent antibodies. The MENA region also sees the same trend of replacing traditional cell culture with shell-vial cultures. In Israel, shell-vial culture is the method in use for the diagnosis of congenital CMV from urine specimens from the infants [72,89,102,118,143,144].

While we summarized in this review the techniques used in CMV diagnosis, they do not necessarily reflect the status of CMV diagnosis in the region. The reviewed studies did not cover all the countries in the MENA region; thus, a more in-depth analysis was not possible. Nevertheless, the information does reflect the availability of the diagnostic tools in the region during the study period. However, even though the diagnostic tools are available and widely used in the region, there is no information on country-wide screening guidelines and policies. Instead, hospitals implement their own practices and guidelines on screening.

## 5. Conclusions

The literature search yielded a total of 72 primary research articles covering 11 out of the 21 MENA countries over 31 years. The studies on transplant and transfusion recipients reported the incidence of CMV with uncertainty about the disease. Although infection with CMV virus is common among graft recipients in this region (up to 77%), the actual rate of clinically confirmed CMV disease was unclear as most of the studies depend solely on seroprevalence reports. Multiple other studies exist outside of those included in this review; however, they do not measure the incidence of CMV. Although some studies assessed the frequency of transplantation, the CMV incidence as a critical outcome was not reported in many of the studies. Similarly, with blood transfusion, studies report CMV seroprevalence with no clear indication of clinical burden. The same can be observed with cCMV, where substantial variability in cCMV reporting criteria leads to a less-than-accurate picture about the status of cCMV in the MENA region. The low number of published articles, the low number of reporting countries, the inconsistency in measurement and detection protocols, as well as the variability in endpoint evaluation of the clinical illness, are all factors that limited the ability to generalize the information across the region. Thus, there is an incessant need to conduct well-designed studies under the umbrella of the WHO to estimate the burden of CMV disease on transplant recipients in the MENA region and to standardized both the prophylactic and preemptive treatment and predict the subsequent morbidity and mortality.

## Figures and Tables

**Table 1:** Cytomegalovirus (CMV) in transplant and transfusion recipients in the MENA region.

Country	Study Type	Study Period	Transplant Type	No. of Patients	Seroprevalence	Median Time of Detection after TX	Symptoms and Complications	Reference
Iran	Retrospective study	1984–2002	Kidney	1925	5.2%	1 to 9 months	Elevated serum creatinine, fever, thrombocytopenia, nausea, vomiting, elevated alkaline phosphatase, leukocytosis, leukopenia, rarely pneumonia, conjunctivitis, vascular dermatitis.	Pour-Reza-Gholi et al., 2005 [28]
Iran	Retrospective study	1984–2007	Kidney	2211	2.1%	NM	1 patient died, 3 lost their allograft function	Nemati et al., 2008 [20]
Iran	Cross-sectional study	1991–2010	Kidney	96	37.5%	NM	NM	Khameneh et al., 2013 [57]
Tunisia	Cohort study	1994–1998	Kidney	18	72%	First 30 days	Six patients had acute rejection	Charfeddine et al., 2002 [21]
Iran	Retrospective study	1996–2007	Liver	22	4.5%	NM	NM	Yaghobi et al., 2010 [58]
Saudi Arabia	Cross-sectional	1996–2014	Donors	263	13.2%	NM	NM	Alsuhaibani et al., 2015 [51]
Iran	Retrospective study	1998–2014	Kidney	725	24.6%	First 5 months	Weakness, fever, respiratory symptoms	Babazadeh et al., 2017 [27]
Kuwait	Cohort Study	2000–2005	Kidney	54	11.11%	NM	NM	Madi et al., 2011 [59]
Saudi Arabia	Retrospective study	2000–2006	Kidney	689	3.6%	NM	Fever, malaise, leukopenia	Basri et al., 2007 [60]
Iran	cohort study	2001–2002	BMT	15	53.5%	4 weeks	Fever; gastrointestinal; skin lesion; retinitis, pneumonia, UTI	Behzad-Behbahani et al., 2004 [24]
Egypt	Retrospective study	2001–2003	BMT	28	39%	NM	NM	Zekri et al., 2004 [42]
Iran	Cross-sectional	2002–2004	Kidney	179	17.6%	NA	Fever, malaise, arthralgias, myalgia, leukopenia and/or thrombocytopenia, or tissue-invasive disease	Pourmand et al., 2007 [61]
Iran	Retrospective study	2002–2006	BMT	104	IgG seroprevalence: 8.7% IgM Seroprevalence: 9.6%	1st–10th weeks	NA	Ramzi et al., 2009 [62]
Saudi Arabia	Retrospective study	2002–2007	Cord blood	73	58.9%	NM	NM	Al-Hajjar et al., 2011 [34]
Israel	Cohort study	2003	Liver	81	8.5%	NA	Fever, disturbed liver functions in all patients, one patient had concurrent CMV pneumonitis and one CMV retinitis	Shibolet et al., 2003 [29]
Iran	Retrospective study	2005–2008	Kidney	925	IgG seroprevalence: 100%	NA	NA	Saghafi et al., 2009 [63]
Saudi Arabia	Retrospective study	2005–2011	HSCT	82	1.22%	NA	NA	Al-Sweedan et al., 2017 [64]
Jordan	Retrospective study	2005–2013	HSCT	72	31%	23 days (12–31) post-transplantation	None of the patients developed CMV disease	Hussain et al., 2015 [65]
Sudan	Retrospective study	2006	Kidney	98	32.7%	2–3 months after kidney transplantation	Fever, diarrhea, hepatitis, neutropenia and/or thrombocytopenia.	Enan et al., 2011 [25]
Iran	Prospective study	2006–2008	Kidney	40	Infection ^a^: 82.5% Disease ^b^: 25%	Infection: 4.7 weeks Disease: 11 weeks	CMV disease, nine patients manifested with elevated serum creatinine values and one, elevated liver enzymes	Taherimahmoudi et al., 2009 [41]
Iran	Retrospective study	2006–2013	Liver	145	32%	12 to 445 days post transplantation	Only 1 patient (2%) developed CMV disease	Davoudi et al., 2014 [66]
Oman	Retrospective study	2006–2015	Kidney	703	14.5%	21 months (15 days–84 months)	Fever, diarrhea, pneumonitis, lymphopenia, anemia, thrombocytopenia	Siddiqui et al., 2017 [26]
Iraq	Cross-sectional study	2007–2008	Kidney	43	97.7%	NA	NA	Al-Alousy et al., 2011 [67]
Iran	Cross-sectional	2007–2010	Transfusion	96	IgG Seroprevalence: 77.4% IgM seroprevalence: 7.1%	NA	NA	Sepehrvand et al., 2010 [54]
Israel	Retrospective study	2007–2012	HSCT	121	61%	NA	First CMV infection with myeloablative conditioning and acute GVHD	Cohen et al., 2015 [22]
Iran	Prospective study	2008	Kidney	68	70.6%	NA	19 cases of acute rejection	Khameneh et al., 2008 [68]
Iran	Cross section	2009–2010	Kidney	91	34.4%	30 days	NA	Nasiri et al., 2011 [69]
Iran	Retrospective study	2011–2013	HSCT	126	34%	40 days (3–77) after transplantation	GI, dermal symptoms with hepatic involvement * 9 cases develop GVHD	Valadkhani et al., 2016 [23]
Iran	Case-control study	2012–2013	coronary artery bypass graft (CABG)	110	CMV DNA in Cases: 14.5% CMV DNA in Controls: 4%	NM	CMV in aortic plaques associated with increased risk of atherosclerosis	Heydar et al., 2015 [30]
Iran	Cross-sectional	2012–2013	Kidney Graft	96	19.8%	NM	NM	Khameneh et al., 2013 [57]
Iran	Cross-sectional	2012–2013	Donors	1008	IgG seroprevalence: 99.2% IgM seroprevalence: 1.6%	NA	NA	Safabakhsh et al., 2013 [53]
Kuwait	Retrospective study	2012–2014	Kidney	1168	15.4%	NA	41% have graft rejection, 34.4% develop systemic CMV disease, 24.5% develop CMV syndrome, 1.6% died	Madi et al., 2015 [19]
Iran	Cohort study	2013	Kidney	82	49%	Four months post-transplantation	The study aimed to correlate CMV infection with decreasing in vitamin D level.	Saber et al., 2015 [70]
Egypt	Cross-sectional	2016	Donors	88	IgG Seroprevalence: 96.6%	NA	NA	Gawad et al., 2016 [56]

* NM: Not mentioned; NA: Not applicable, due to the subjects being healthy individuals. ^a^: Infection was defined by the presence of anti-CMV IgG, anti-CMV IgM, CMV DNA, or a positive result for the pp65 antigenemia assay. ^b^: Disease was defined by the presence of symptoms.

**Table 2:** CMV in the MENA region.

Country	Study Type	Study Period	No. of Patients	CMV Results	Symptoms and Complications	Reference
Kuwait	Experimental study	1988	575 infants	2.6% positive IgM	NM	El-Mekki et al., 1988 [101]
Israel	Retrospective study	1993–1997	63 pregnant women	34.8% showed vertical transmission	Abnormal ultrasound, neurologic sequelae	Lipitz et al., 1997 [76]
Iraq	Prospective-follow up until delivery	1999	60 pregnant women	10% CMV IgM in cord blood	Congenital malformations, microcephaly	Al-Ali et al., 1999 [86]
Israel	Retrospective study	1999–2008	59 primary Periconceptional CMV infection	18.6% CMV infections	NM	Hadar et al., 2010 [73]
Israel	Retrospective study	2001–2012	9845 infants	0.57% CMV infection	Abnormal hearing	Barkai et al., 2014 [81]
Iran	case-control study	2002–2003	95 with sensory hearing loss	34.6% CMV infection	Sensorineural hearing loss	Pasternak et al., 2018 [102]
Iran	This case-control study	2003–2004	250 women with a history of abortion and 200 matched with no abortion	5% positive for CMV	Abortion	Jahromi et al., 2010 [91]
Israel	A prospective study	2005	70 infants who received breast milk from seropositive mothers	5.7% acquired CMV by the second or third week of pregnancy	NM	Miron et al., 2005 [103]
Israel	Experimental study	2005	5000 Newborns	81.5–85% serum IgM 0.7% had cCMV infection	NM	Ziyaeyan et al., 2007 [97]
Israel	Retrospective study	2005–2013	149	36% CMV infection	Severe hearing loss	Bilavsky et al., 2016 [104]
Israel	Retrospective-cohort study	2005–2012	210 infants with cCMV	75% symptomatic 25% asymptomatic	Prematurity, abnormal hearing, lenticulostriate vasculopathy	Bilavsky et al., 2015 [72]
Israel	Retrospective study	2005–2013	284 infants with cCMV	69.7% symptomatic 30.3% asymptomatic	Hepatitis, cholestatic disease	Bliavsky et al., 2015 [88]
Palestine	Retrospective study	2006–2012	249 newborns	4 out of 249 newborns with cCMV born to mothers with positive CMV DNA in urine	NM	Neirukh et al., 2013 [105]
Israel	Retrospective case-control study	2006–2015	138 infants with cCMV	66.67% positive with amniocentesis	Abnormal hearing, developmental delay	Bilavsky et al., 2016 [106]
Iran	Experimental study	2007	92 pregnant women with caesarian section	98% of women had positive serum IgG 5.4% of women had positive serum IgM Neonates from IgG positive mothers had positive IgM	NM	Townsend et al., 2013 [107]
Iran	Experimental study	2008	844 pregnant women	93% had positive serum IgG 5% had positive serum IgM	Congenital disorders	Arapour et al., 2008 [83]
Israel	Observational study	2008	Twenty-eight pregnant mothers primary CMV infection acquired after 25 weeks of gestation	21 neonates had a vertical transmission with no symptoms One pregnancy was terminated in 36 weeks with apparent symptoms	All 21 infected neonate showed clinical symptoms of CMV infection	Gindes et al., 2008 [75]
Israel	Retrospective study	2009	All pregnant mothers with positive IgM & high IgG Avidity	79 women with CMV IgM-high IgG avidity combination (indicate past infection)	NM	Kanengisser -Pines et al., 2009 [108]
Israel	Retrospective study	2009–2010	8105 infants	0.28% prevalence	CNS involvement, abnormal hearing	Barkai et al., 2013 [82]
Israel	Cohort study	2010	27 cCMV infected fetuses	Temporal lobe volumes were significantly smaller in fetuses infected with CMV compared to uninfected fetuses	Severe brain dysmorphology in first and second trimesters.	Hoffman et al., 2010 [74]
Egypt	Experimental study	2011	33 neonate and mothers	Four neonates with positive IgM, two of which had mothers with positive IgM	Gastrointestinal complications	Abu Faddan et al., 2011 [109]
Israel	Prospective study behavioral studies of LSV symptoms	2011	92 infants with congenital CMV	50 cases had lenticulostriate vasculopathy and hearing loss.	CNS impairment, abnormal hearing	Amir et al., 2011 [80]
Israel	Retrospective study	2011	Infected infants (CMV DNA positive)	NM	Abnormal white matter	Farkas et al., 2011 [78]
Oman	Retrospective review	2011–2012	373 infants	34 positives cases	Death, prolonged PICU stay, respiratory complication	Abdelmogheth et al., 2014 [90]
Kuwait	Prevalence study-follow up until pregnancy	2013	983 pregnant mothers	9% positive cord blood IgM 0.9% positive urine PCR. Seven of the nine cases had a high viral load	NA	Al-Awadhi et al., 2013 [110]
Egypt	Cross-sectional study	2013	546 pregnant women	100% positive serum IgG 7.3% positive serum IgM with an intermediate IgG avidity index. Of these, 50% had higher avidity indices after the 3rd trimester.	NM	Kamel et al., 2014 [111]
Israel	Prospective cohort study	2013	142 pregnant women with primary CMV infection and vertical transmission in the 1st and second trimesters	The primary infection occurred in the 1st (50%) and second (50%) trimester Seven pregnancies terminated with neurologic sequelae one neonate died due to neurologic complications	Auditory damage or neurodevelopmental disabilities	Lipitz et al., 2013 [77]
Iran	Prevalence study	2013–2014	100 symptomatic infants less than 3-weeks old	58% with cCMV	Hearing loss	Ebrahimi-Rad et al., 2017 [96]
Iran	Cross-sectional study	2014	225 pregnant women and their newborns	100% of mothers had positive IgG, of which 2.7% had positive IgM Women with normal deliveries showed low IgG compared to caesarian section	CMV infection by radiological evaluation (CT scan)	Erfanianahmadpoor et al., 2014 [112]
Israel	Retrospective study	2014–2015	178 infants with hearing disability	2.2% with cCMV	CNS symptoms	Ari-Even Roith et al., 2017 [79]
Iran	Prospective study	2014–2016	1617 neonate	0.49% with cCMV	Short-term growth impairment	Karimian et al., 2016 [84]
Sudan	Experimental study	2015	50 infants	8% with cCMV	Congenital anomalies	Ebrahimet al., 2015 [113]
Sudan	Experimental study	2016	90 pregnant women	98.9% had positive serum IgG 1.1% had positive serum IgM	NA	Altayeb et al., 2016 [114]
Israel	Retrospective cohort study	2016	98 infants from infected mothers	52 received antiviral upon delivery	Lenticulostriate vasculopathy on postnatal US, Sensorineural hearing loss	Amir et al., 2016 [71]
Iran	Case control study	2016	81 pregnant women who aborted	Anti-CMV IgM was higher compared to controls (25.9% compared to 12.2%; OR = 12.2, *p* = 0.019)	Early abortion	Rasti et al., 2016 [85]
Israel	Retrospective cohort study	2017	107 infants with cCMV 95 of which are from mothers with primary infection, 12 from mothers with non-primary infection	Incidence of abnormal brain sonographic findings high in infants of mothers with primary infection was 67%, compared to non-primary infection 8.3%	Infant’s Mothers acquired gestational hypertensive disorder and GDM	Hadar et al., 2010 [89]
Iraq	This prospective study	2019	24 neonates	96% with CMV infection	Jaundice-, hepatosplenomegaly	Alwan et al., 2019 [115]
Iran	Pilot study	January 2012 to March 2012	620 infants	0.32% positive for CMV DNA	Infected infants showed no symptoms	Fahimzad et al., 2013 [87]

* NM: Not mentioned; NA: Not applicable, due to the subjects being healthy individuals; cCMV: Congenital CMV; EP: Ectopic pregnancy; CNS: Central nervous system; FT: Liver function; RF: Rheumatoid factor; CT: Computed tomography; GDM: Gestational diabetes mellitus.
